# Honokiol Arrests Cell Cycle, Induces Apoptosis, and Potentiates the Cytotoxic Effect of Gemcitabine in Human Pancreatic Cancer Cells

**DOI:** 10.1371/journal.pone.0021573

**Published:** 2011-06-24

**Authors:** Sumit Arora, Arun Bhardwaj, Sanjeev K. Srivastava, Seema Singh, Steven McClellan, Bin Wang, Ajay P. Singh

**Affiliations:** 1 Department of Oncologic Sciences, Mitchell Cancer Institute, University of South Alabama, Mobile, Alabama, United States of America; 2 Department of Mathematics and Statistics, College of Arts and Sciences, University of South Alabama, Mobile, Alabama, United States of America; 3 Department of Biochemistry and Molecular Biology, College of Medicine, University of South Alabama, Mobile, Alabama, United States of America; Roswell Park Cancer Institute, United States of America

## Abstract

Survival rates for patients with pancreatic cancer are extremely poor due to its asymptomatic progression to advanced and metastatic stage for which current therapies remain largely ineffective. Therefore, novel therapeutic agents and treatment approaches are desired to improve the clinical outcome. In this study, we determined the effects of honokiol, a biologically active constituent of oriental medicinal herb *Magnolia officinalis/grandiflora*, on two pancreatic cancer cell lines, MiaPaCa and Panc1, alone and in combination with the standard chemotherapeutic drug, gemcitabine. Honokiol exerted growth inhibitory effects on both the pancreatic cancer cell lines by causing cell cycle arrest at G_1_ phase and induction of apoptosis. At the molecular level, honokiol markedly decreased the expression of cyclins (D1 and E) and cyclin-dependent kinases (Cdk2 and Cdk4), and caused an increase in Cdk inhibitors, p21 and p27. Furthermore, honokiol treatment led to augmentation of Bax/Bcl-2 and Bax/Bcl-xL ratios to favor apoptosis in pancreatic cancer cells. These changes were accompanied by enhanced cytoplasmic accumulation of NF-κB with a concomitant decrease in nuclear fraction and reduced transcriptional activity of NF-κB responsive promoter. This was associated with decreased phosphorylation of inhibitor of kappa B alpha (IκB-α) causing its stabilization and thus increased cellular levels. Importantly, honokiol also potentiated the cytotoxic effects of gemcitabine, in part, by restricting the gemcitabine-induced nuclear accumulation of NF-κB in the treated pancreatic cancer cell lines. Altogether, these findings demonstrate, for the first time, the growth inhibitory effects of honokiol in pancreatic cancer and indicate its potential usefulness as a novel natural agent in prevention and therapy.

## Introduction

Pancreatic cancer is one of the most lethal malignancies in the United States with mortality rate increasing every coming year [1], [2]. According to the estimate of American Cancer Society, 43140 Americans were diagnosed with pancreatic cancer in 2010 and 36,800 died, marking this malignancy as the fourth leading cause of death from cancer [2]. Due to its asymptomatic progression, pancreatic cancer is diagnosed at a stage when it has already metastasized or is locally advanced [3]. Therapeutic approaches against the advanced disease have largely failed and approximately >80% of patients diagnosed with this malignancy still die within 2–8 months [4]. Gemcitabine, a standard FDA approved drug for pancreatic cancer therapy, is reported to be minimally effective that improves patient's survival by couple of weeks only [3], [5]. Therefore, it is of utmost importance to develop alternative therapeutic regimens and strategies for effective management of pancreatic cancer.

Several new strategies, which target growth promoting pathways alone and in combination with gemcitabine, have been tested in pancreatic cancer to improve therapeutic outcome [6]. In addition, several recent studies have identified deregulated signaling elements, such as Ras, Akt, NF-κB, miRNAs, etc., that not only promote cancer progression but also confer chemoresistance in pancreatic cancer [7]–[9]. Induction of these survival pathways results from activating gene mutations, loss of inhibitory pathways and/or potentiation through autocrine and paracrine signaling mechanisms [3], [10]. In fact, it has now been shown that targeting of some of these signaling nodes can be useful in inhibiting tumor growth and progression as well as in restoring the sensitivity of tumor cells to the cytotoxic drugs [3], [9], [10].

Natural products have been at the core of cancer chemotherapy for past several decades and in fact, over 60% of the current anticancer drugs have their origin from natural sources [11]. In several recent studies, novel plant-derived compounds have been identified to act as anti-tumor agents through modulation of biological pathways [12]. Honokiol, a biologically active biphenolic compound isolated from the *Magnolia officinalis/grandiflora,* has received significant attention due to its potent anti-neoplastic and anti-angiogenic properties [13], [14]. It has yielded promising data against skin, colon, lung and breast cancers [13], [15]–[17]. The striking aspect of honokiol as an anti-neoplastic drug is its potential to inhibit nuclear factor kappa B (NF-κB), which is associated with cancer cell survival and chemoresistance [9], [10], [18]. The NF-κB is constitutively activated in a variety of hematologic and solid malignancies, including pancreatic cancer and controls the expression of an array of genes involved in cell proliferation and survival through direct and indirect mechanisms [18]–[20]. In the present study, we have examined, for the first time, the effects of honokiol against pancreatic cancer. Our data show that honokiol inhibits the growth of human pancreatic cancer cell lines, MiaPaCa and Panc1, by causing cell cycle arrest and induction of apoptosis. Furthermore, our study provides evidence for a role of honokiol in chemosensitizing the pancreatic cancer cells to cytotoxic effects of gemcitabine.

## Results

### Growth inhibitory effect of honokiol on human pancreatic cancer cells

**Figure 1:**
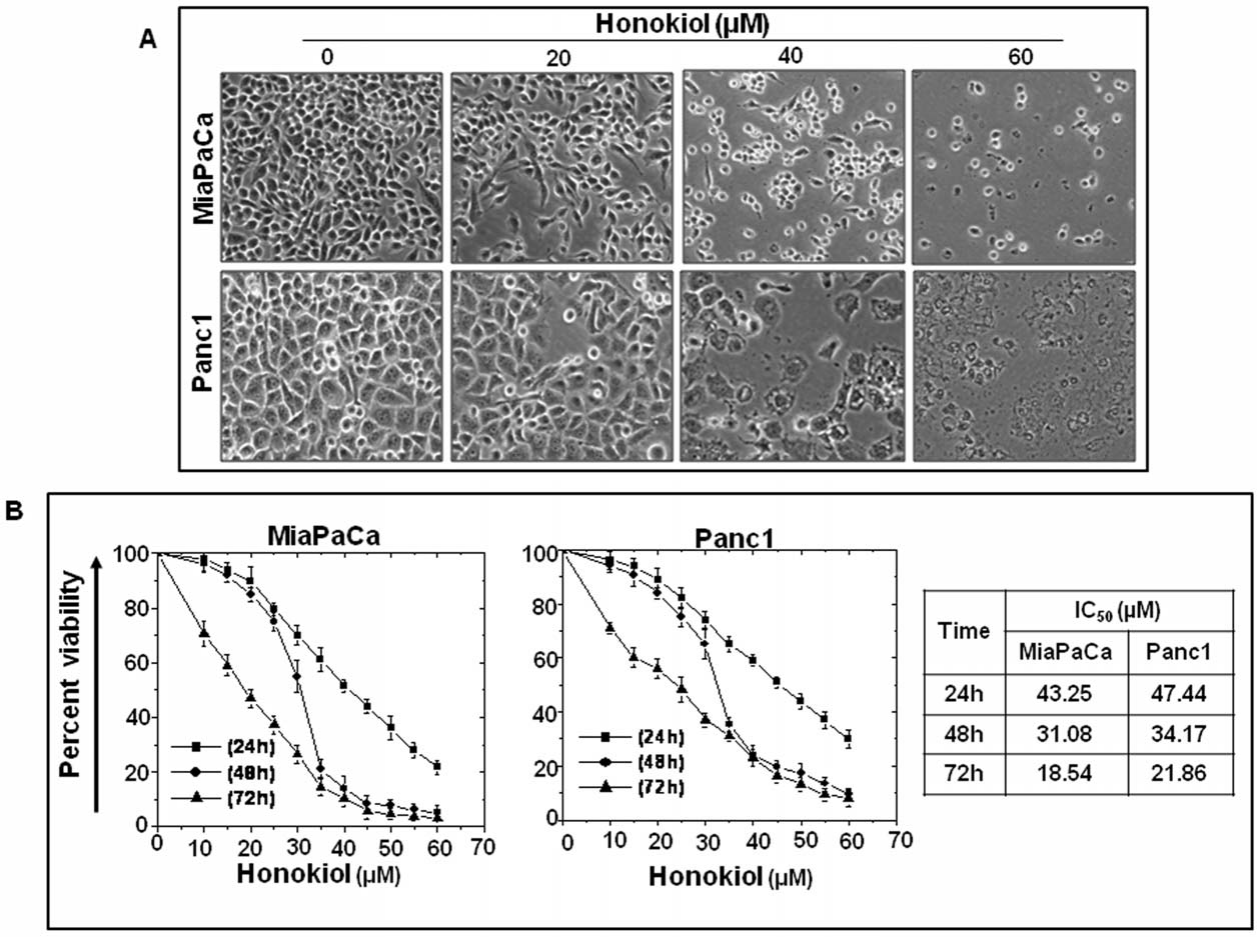
Honokiol suppresses growth of human pancreatic cancer cells. (**A**) MiaPaCa and Panc1 cells were seeded in 6 well plate (1×10^5^ cells/well) and allowed to attain 70–80% confluence prior to honokiol (10–60 µM) treatment for 48 h. Following treatment, significant change in cell morphology was observed of both the cell types as examined under phase-contrast microscope. Cells became round, shrunken and detached from cell surface in a dose-dependent manner. Representative micrographs are from one of the random fields of view (magnification 200X) of cells treated with 20, 40 or 60 µM honokiol. (**B**) MiaPaCa and Panc1 cells were grown in 96 well microtitre plates (1×10^4^ cells /well) and treated with honokiol (10–60 µM) at 70–80% confluence. Percent viability of cells was measured by WST-1 assay after 24, 48 and 72 h. An OD value of control cells (treated with an equal volume of DMSO, final concentration, <0.1%) was taken as 100% viability. Honokiol inhibited cell viability in a dose- and time- dependent manner for both the cell types suggesting anti-tumor effect of honokiol. Data are expressed as mean± SD; (n = 3).

Two human pancreatic cancer cell lines viz. MiaPaCa and Panc1 were employed as a model system to investigate the effect of honokiol on pancreatic cancer cell growth. Cells treated with honokiol (10–60 µM) showed alterations in morphology as compared to vehicle (DMSO)-treated cells. With increasing concentration of honokiol, cells became round, shrunken and detached from the substratum (Figure 1A), consistent with morphological changes associated with apoptosis. Subsequently, we quantified the cytotoxic effects of honokiol by measuring percent viability using WST-1 assay. Our data demonstrated that honokiol induced a dose- and time- dependent decrease in growth of both the pancreatic cancer cell lines with IC_50_ values of ∼43.25, 31.08 and 18.54 µM (against MiaPaCa), and ∼47.44, 34.17 and 21.86 µM (against Panc1) after 24, 48 and 72 h treatments, respectively (Figure 1B). Together, these findings indicate that honokiol has growth inhibitory effects on pancreatic cancer cells.

### Honokiol causes G_1_ phase cell cycle arrest and induces apoptosis in pancreatic cancer cells

**Figure 2:**
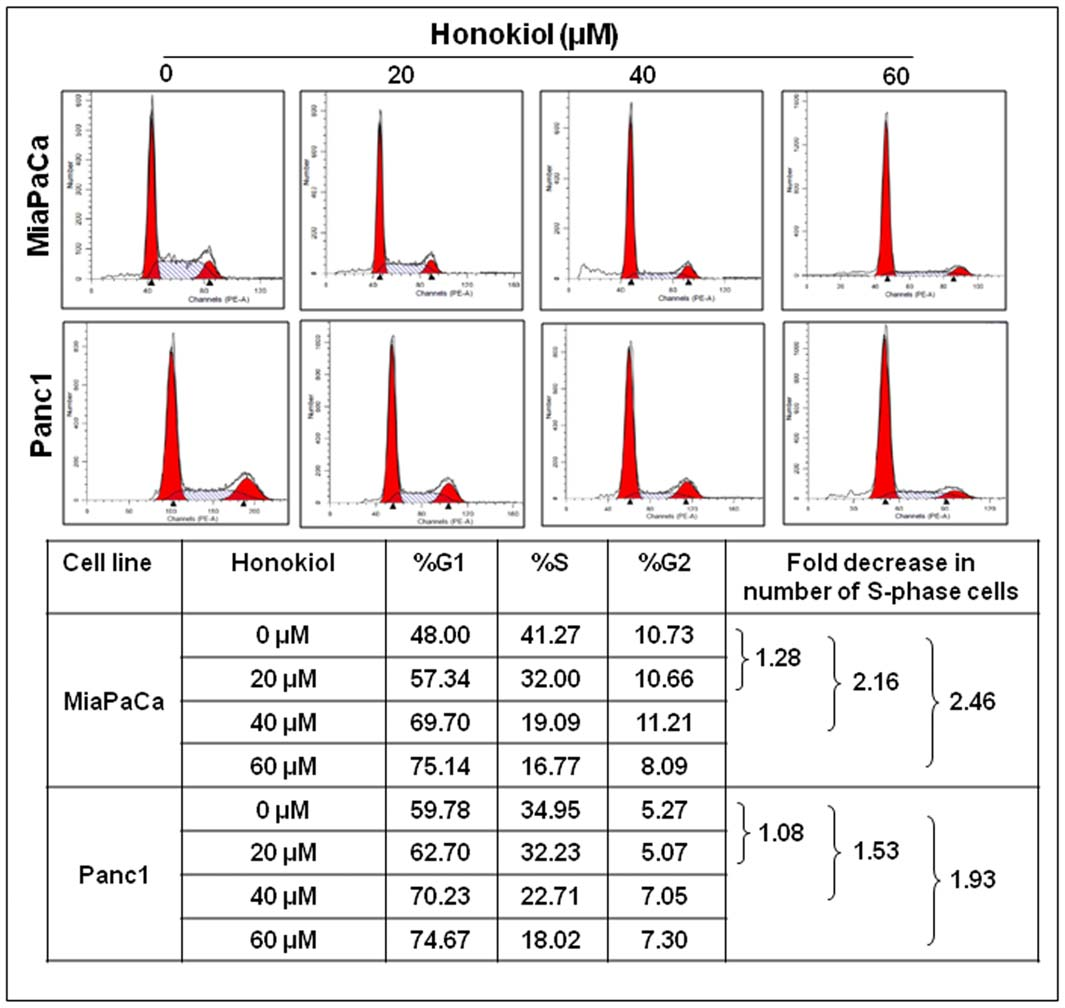
Honokiol causes G_1_ phase cell cycle arrest in human pancreatic cancer cells. MiaPaCa and Panc1cells (1×10^6^ cells/well) were synchronized by culturing in serum free media for 72 h, followed by incubation in serum-containing media for 24 h and subsequent treatment with either honokiol (20, 40 or 60 µM) or DMSO (control) for 24 h. Distribution of cells in different phases of cell cycle was analyzed by propidium iodide (PI) staining followed by flow cytometry. Enhanced accumulation of MiaPaCa and Panc1 cells in the G_1_ phase of the cell cycle was observed after treatment with honokiol in a dose-dependent manner (as indicated by flow histograms) with a concomitant decrease in S-phase cells.

**Figure 3:**
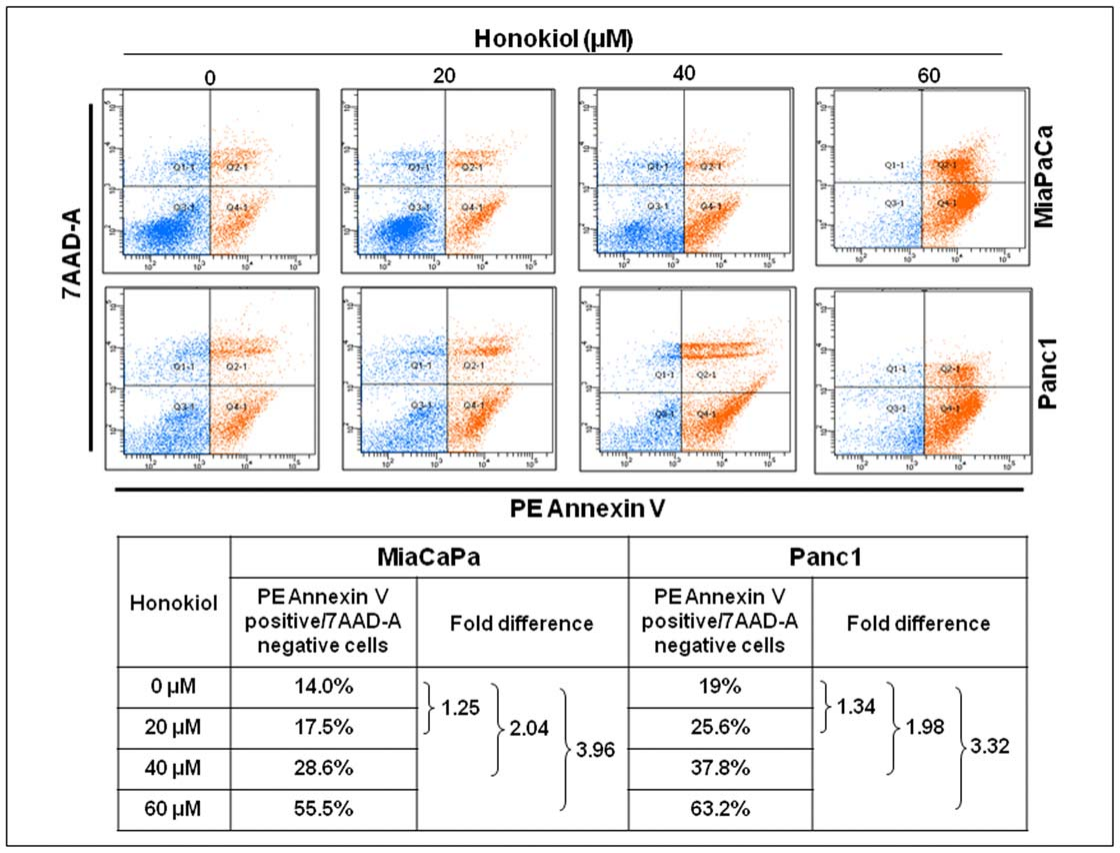
Honokiol induces apoptosis in human pancreatic cancer cells. MiaPaCa and Panc1 cells were grown in 6-well plates (1×10^6^ cells /well) and allowed to attain 70–80% confluence. Cells were treated with either honokiol (20, 40 or 60 µM) or DMSO (control) for 24 h and subsequently stained with 7-AAD and PE Annexin V followed by flow cytometry. The lower left quadrants of each panels show the viable cells (negative for both, PE Annexin V and 7-AAD). The upper right quadrants contain necrotic or late apoptotic cells (positive for both, PE Annexin V and 7-AAD). The lower right quadrants represent the early apoptotic cells (PE Annexin V positive and 7-AAD negative). Data show a dose-dependent increase in the number of apoptotic cells in both MiaPaCa and Panc1 cells after treatment with honokiol as compared to control cells, indicating apoptotis inducing potential of honokiol.

Suppression of cancer cell growth can be caused either by arrest of cell cycle progression or due to induction of apoptosis or both [12]. Our data on cell cycle distribution demonstrated that treatment with honokiol resulted in enrichment of pancreatic cancer cells in G_1_ phase with a concomitant decrease in number of cells in S-phase (proliferative fraction) (Figure 2). We observed a ∼1.28, 2.16 and 2.46 folds (in MiaPaCa) and ∼1.08, 1.53 and 1.93 folds (in Panc1) decrease in number of cells in S-phase at 20, 40 and 60 µM doses of honokiol, respectively (Figure 2). In apoptosis assays, our data demonstrated a considerable increase in apoptotic index (PE Annexin V positive/7AAD negative cells) in a dose-dependent manner after 24 h of honokiol treatment (Figure 3). At 20, 40 and 60 µM concentrations of honokiol, we observed ∼1.25, 2.04 and 3.96 folds increase in apoptotic indices of MiaPaCa and ∼1.34, 1.98 and 3.32 folds increase in apoptotic indices of Panc1 cells, respectively. Altogether, our findings demonstrate that honokiol has both cytostatic and cytotoxic properties against pancreatic cancer cells.

### Honokiol alters the expression of cell-cycle and survival-associated proteins

**Figure 4:**
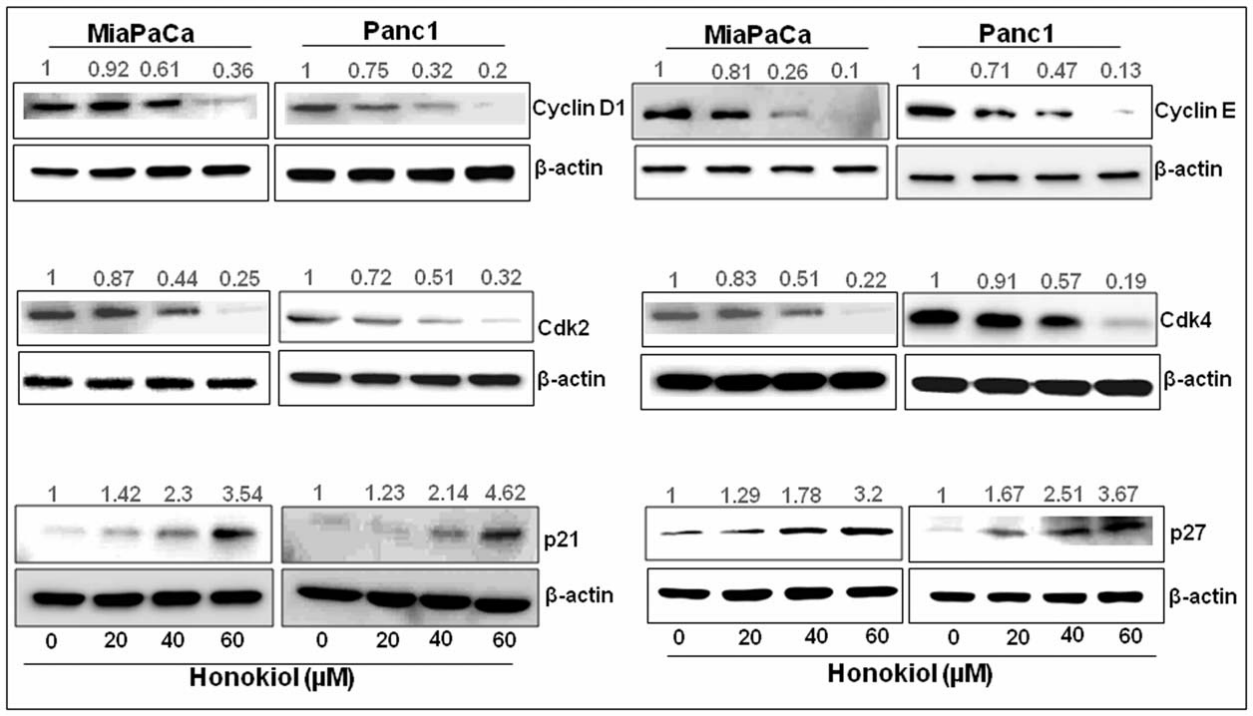
Honokiol treatment leads to altered expression of cell cycle-related proteins in human pancreatic cancer cells. Pancreatic cancer cells (MiaPaCa and Panc1) were treated with either honokiol (20, 40 or 60 µM) or DMSO (control) for 24 h. Total protein was isolated and subjected to immunoblot analysis for various cell cycle-associated proteins (cyclin D1, cyclin E, Cdk2, Cdk4, p21 and p27). β-actin was used as a loading control. Intensities of the immunoreactive bands were quantified by densitometry. Normalized densitometric values are indicated at the top of the bands exhibiting a dose-dependent decrease in the expression of cyclin D1, cyclin E, Cdk2 and Cdk4 and increase in the expression of cyclin inhibitors; p21 and p27, after exposure to honokiol, in both the cell types.

**Figure 5:**
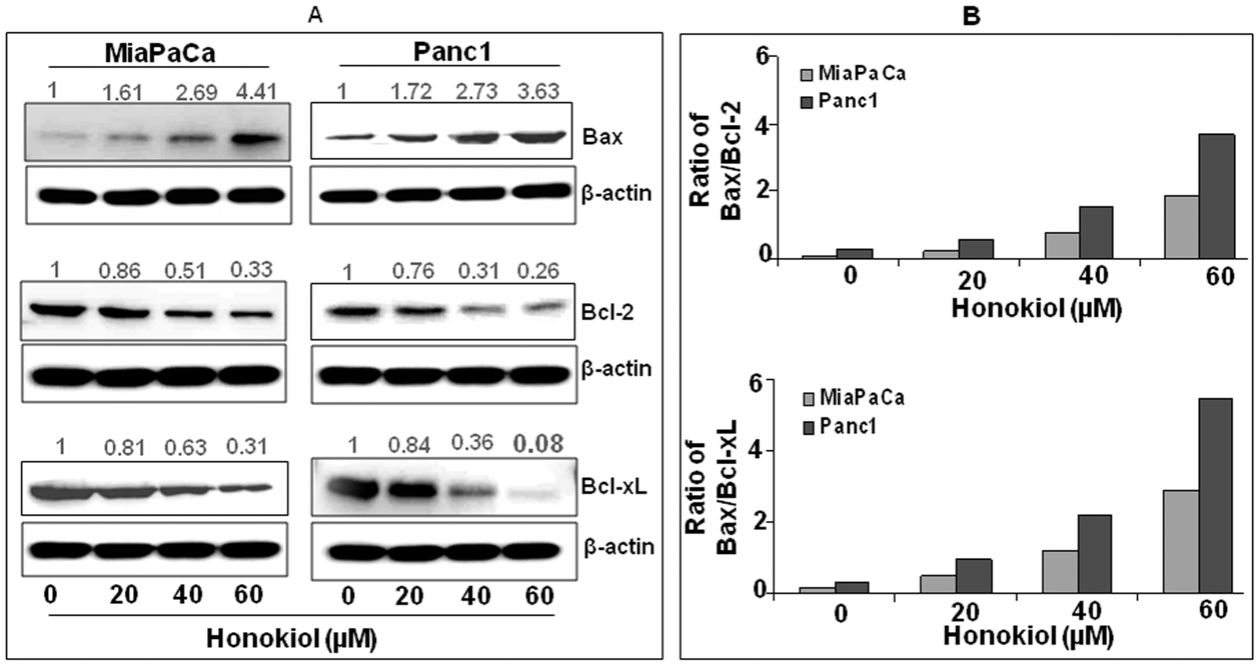
Honokiol modulates Bax/Bcl-2 and Bax/Bcl-xL ratio in human pancreatic cancer cells. (A) MiaPaCa and Panc1 cells were treated with either honokiol (20, 40 or 60 µM) or DMSO (control) for 24 h. Immunoblotting was performed for Bcl-xl, Bcl-2 and Bax proteins followed by densitometry of immunoreactive bands. Normalized densitometric values are indicated at the top of the bands. (B) Bar diagram summarizing the effects of honokiol treatment on Bax/Bcl-2 ratio (upper panel) and Bax/Bcl-xL ratio (lower panel). Data suggest that honokiol induces apoptosis by upregulating pro-apoptotic Bax and downregulating anti-apoptotic Bcl-2 and Bcl-xL proteins.

To investigate the mechanistic basis of growth inhibitory effects of honokiol, we next examined its effect on the expression of key proteins involved in cell proliferation and survival. Our data revealed a dose-dependent decrease in the expression of cyclins (D1 and E) and cyclin-dependent kinases (Cdk2 and Cdk4); while an induced expression of cyclin-dependent kinase inhibitors (p21 and p27) was observed after honokiol treatment in both MiaPaCa and Panc1 pancreatic cancer cells (Figure 4). Among the survival proteins, we observed a dose-dependent reduction in the levels of the anti-apoptotic protein Bcl-2 and Bcl-xL, whereas a concomitant increase in the level of pro-apoptotic protein Bax was observed (Figure 5A) leading to an increase in the ratio of Bax/Bcl-2 (Figure 5B, upper panel) and Bax/Bcl-xL (Figure 5B, lower panel). These findings demonstrate that honokiol alters the expression of proteins involved in the regulation of cell cycle and apoptosis to confer its growth inhibitory effect.

### Honokiol attenuates the constitutive activation of NF-κB in human pancreatic cancer cells

**Figure 6:**
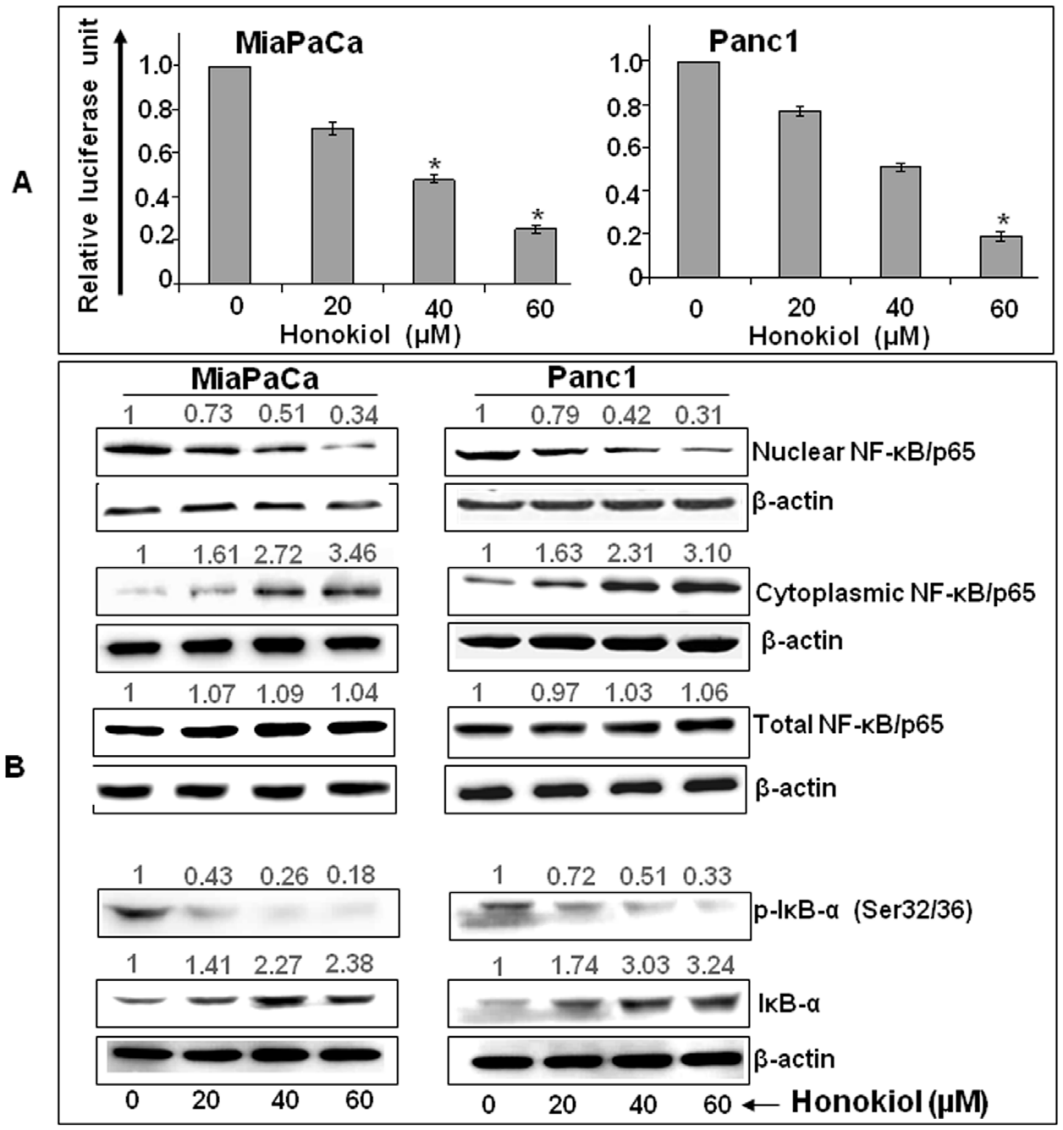
Honokiol attenuates constitutive NF-κB activation by inhibiting nuclear translocation of NF-κB/p65 in human pancreatic cancer cells. (A) MiaPaCa and Panc1cells (0.5×10^6^ cells/well) were seeded in 12-well plate. Next day at 60% confluence, cells were co-transfected with NF-κB luciferase reporter and TK-Renilla luciferase (control) plasmids. Twenty-four hours post-transfection, cells were treated with honokiol (20, 40, or 60 µM) for next 24 h. Protein lysates were made and luciferase (Fire-fly; test and Renilla, transfection efficiency control) activity assessed using a dual-luciferase assay system. Data is presented as normalized fold-change in luciferase activity (mean± SD; n = 3, * p<0.05). (B) Total, nuclear and cytoplasmic extracts were prepared from cells treated with honokiol (20, 40, or 60 µM) for 6 h and expression of NF-κB/p65, p-IκB-α (S32/36) and IκB-α was determined by Western blot analysis. β-actin was used as a loading control. Intensities of the immunoreactive bands were quantified by densitometry. Normalized densitometry values are indicated at the top of the bands indicating a decreased localization of NF-κB/p65 in nucleus with a concomitant increase in cytoplasm. In contrast, expression of p-IκB-α was decreased leading to increased levels of IκB-α. Altogether, these data clearly suggest that honokiol inhibits NF-κB activity through stabilization of IκB-α.

NF-κB is constitutively active in many cancer types, including pancreatic cancer [18]–[20] and it has been shown that the activation of this signaling node facilitates cell cycle progression [21] and apoptotic resistance [22]. Therefore, we investigated whether the treatment of pancreatic cancer cells with honokiol has an impact on NF-κB activation in pancreatic cancer cells. We first examined the effect of honokiol on the transcriptional activity of NF-κB- responsive promoter in a luciferase reporter assay. Our data indicated a dose-dependent reduction in transcriptional activity of NF-κB (∼1.40, 2.08 and 4.0 folds in MiaPaCa, and ∼1.29, 1.96 and 5.26 folds in Panc1 cells) at 20, 40 and 60 µM of honokiol treatment, respectively (Figure 6A). To further support this observation, we next studied the cellular localization (cytoplasmic vs. nuclear) p65 subunit of NF-κB. Our immunoblot data demonstrated that honokiol treatment caused a marked and dose-dependent decrease in NF-κB levels in the nuclear fraction of both MiaPaCa and Panc1 pancreatic cancer cells with a simultaneous increase in the cytoplasmic fraction (Figure 6B). Cellular distribution of NF-κB is controlled by relative expression of its biological inhibitor IκB, which keeps NF-κB sequestered in cytoplasm in an inactive complex [23]. Therefore, we analyzed the cytoplasmic extracts of honokiol-treated pancreatic cancer cells for determination of IκB-α level. Our data demonstrated a dose-dependent increase in the level of the IκB-α upon honokiol-treatment (Figure 6B). This was associated with a concomitant decrease in IκB-α phosphorylation indicating increased stabilization of IκB-α after exposure to honokiol. Altogether, our data indicate that honokiol suppresses constitutive activation of NF-κB in pancreatic cancer cells.

### Honokiol chemosensitizes the pancreatic cancer cells for gemcitabine toxicity

**Figure 7:**
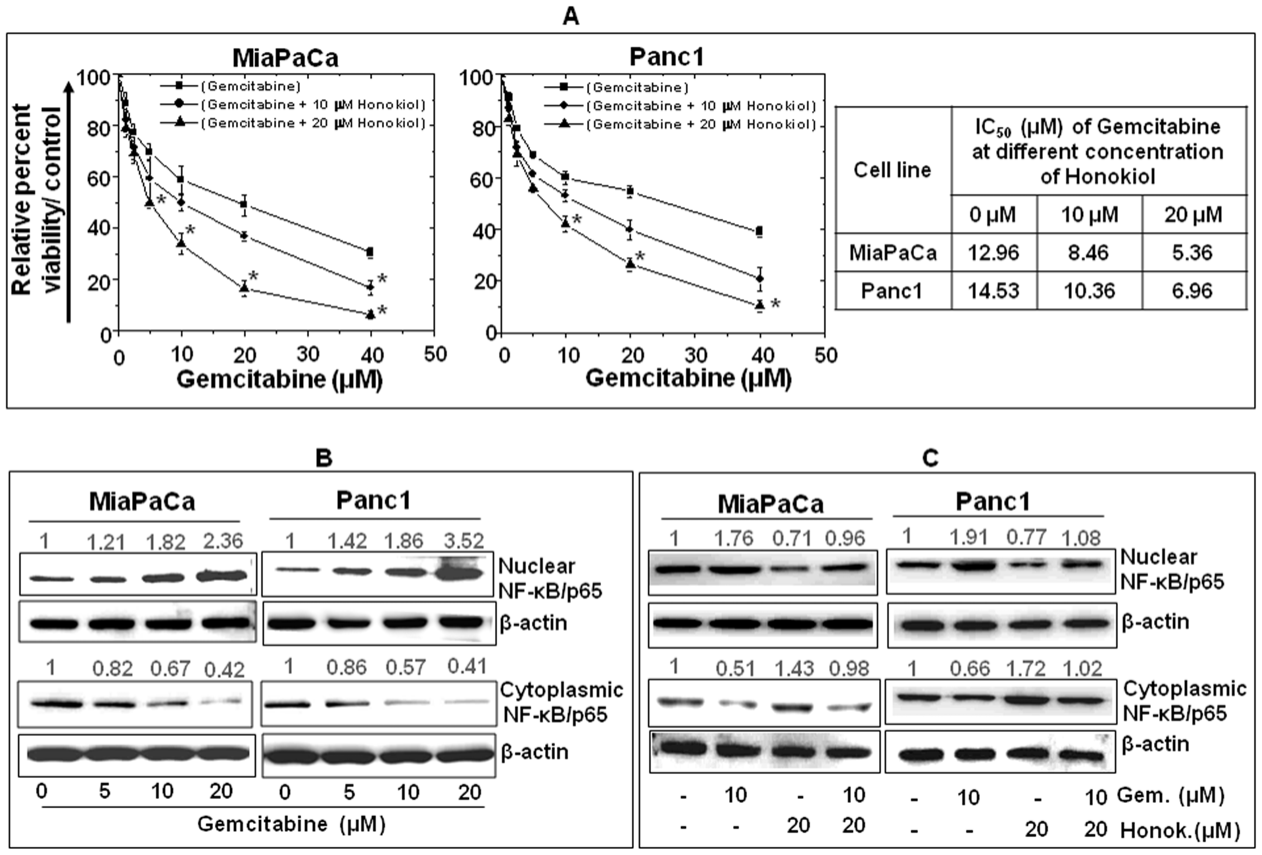
Honokiol chemo-sensitizes pancreatic cancer cells to gemcitabine treatment. (**A**) MiaPaCa and Panc1 cells were grown in 96 well microtitre plates (1×10^4^ cells/well). At sub-confluence, cells were treated with gemcitabine (1.25–40 µM) alone or in combination with honokiol (10 or 20 µM) for 48 h. Percent viability was measured by WST-1 assay. Data are presented as relative percent viability with respect to untreated or honokiol only-treated cells to control for the growth suppressive effects of honokiol (mean± SD; n = 3. *, p<0.05). A significant decrease in IC_50_ values of gemcitabine was observed in cells co-treated with honokiol. (**B**) Cells were treated with gemcitabine (5, 10 and 20 µM) for 6 h and expression of NF-κB/p65 in nuclear, cytoplasmic and total cell lysates was determined by Western blot analysis. Gemcitabine treatment of cells resulted in enhanced nuclear accumulation of NF-κB/p65 in a dose dependent manner (**C**) Nuclear and cytoplasmic extracts were prepared from cells treated with gemcitabine (10 µM), honokiol (20 µM) alone or in combination for 6 h. Expression of NF-κB/p65 in nuclear, cytoplasmic and total cell lysates was determined by Western blot analysis. Data shows that honokiol co-treatment restricted gemcitabine-induced NF-κB/p65 nuclear accumulation. Together, these findings suggest that honokiol acts as a chemosensitizer and increases the cytotoxic effects of gemcitabine in pancreatic cancer.

Gemcitabine is the only FDA-approved chemotherapeutic drug against pancreatic cancer; however, it remains minimally effective due to chemoresistance [3], [5], [10]. Since activation of NF-κB is considered as one of the mechanisms potentiating chemoresistance, we examined if honokiol would act as a chemosensitizer in pancreatic cancer cells. Pancreatic cancer cells (MiaPaCa and Panc1) were treated with gemcitabine alone or in combination with to sub-IC_50_ concentrations of honokiol and effect on growth inhibition was examined using cell viability assay. Our data demonstrated that gemcitabine inhibited the growth of pancreatic cancer cells in a dose-dependent manner and combined treatment with honokiol led to a significant reduction in the IC_50_ of gemcitabine (Figure 7A). At 10 and 20 µM doses of honokiol, respectively, a ∼1.53 and 2.41 fold (in MiaPaCa) and ∼1.40 and 2.08 fold (in Panc1) decrease in IC_50_ of gemcitabine was observed signifying the chemosensitizing effect of honokiol (Figure 7A). To identify a role of NF-κB, we examined its cellular localization in gemcitabine (alone or in combination with honokiol)-treated pancreatic cancer cells. Our data displayed an enhanced accumulation of NF-κB in nuclear compartment and a concomitant decrease in cytoplasmic fraction with increasing doses of gemcitabine in both MiaPaCa and Panc1 cells (Figure 7B). Notably, we observed that honokiol (even at 20 µM dose) was effective in inhibiting the gemcitabine-induced activation of NF-κB in both MiaPaCa and Panc1 cells (Figure 7C). These findings clearly suggest that honokiol potentiates the anti-tumor efficacy of gemcitabine by acting as a chemo-sensitizer in pancreatic cancer cells.

## Discussion

Pancreatic cancer remains a devastating malignancy due to lack of effective therapy for treatment [3]. The present study demonstrated that honokiol (a natural biphenolic compound) is effective in suppressing the growth of human pancreatic cancer cells (MiaPaCa and Panc1) due to its cytostatic and cytotoxic properties. Furthermore, our studies provided evidence for a role of honokiol in chemosensitizing the pancreatic cancer cells to gemcitabine toxicity. Honokiol inhibited NF-κB activity and caused altered expression of many cell cycle and survival-associated proteins to confer its growth suppressive and chemosensitizing effects in pancreatic cancer cells.

Deregulated growth in cancer cells is often attributed to loss of control in proliferative and apoptotic pathways [24]. In fact, molecular studies have revealed that the expression of cell cycle regulators and proteins associated with cell survival is frequently altered in multiple human cancers [25]–[27]. Cell cycle is regulated by concerted actions of cyclins, cyclin-dependent kinases (Cdks) and Cdks inhibitors [26], [28]. We observed that the treatment of pancreatic cancer cells with honokiol resulted in G_1_-phase arrest of cell cycle progression, along with reduction in cyclin D1, cyclin E, Cdk2 and Cdk4 and increase in p21 and p27 at the protein level. Cyclin D1 and its catalytic partner Cdk4 dominate in G_1_ phase, whereas, cyclin E and Cdk2 complex regulates the cell-cycle progression from G_1_ to S [26], [28]. Therefore, our findings indicate that the honokiol-induced arrest of pancreatic cancer cells in G_1_ cell cycle phase might be mediated through the downregulation of cyclins and Cdks along with the upregulation of p21 and p27 proteins, which form heterotrimeric complexes with G_1_-S Cdks and cyclins to inhibit their activity [29]. These results are in accordance with earlier studies on the effect of honokiol in human lymphoid leukemia, squamous lung cancer and breast cancer cells [16], [17], [30].

Following G_1_-phase cell cycle arrest, cells may either undergo repair or enter the apoptotic pathway to maintain cellular integrity and elimination of erred/mutated pre-malignant and neoplastic cells [31]. Thus, the induction of apoptosis is one of the protective mechanisms against cancer initiation and progression and cancer cells have often acquired resistance to apoptosis [24]. In the present study, we observed significant induction of apoptosis in honokiol-treated pancreatic cancer cells indicating that honokiol is able to potentiate the apoptotic machinery. Cell survival is maintained by a fine balance of the ratios of pro-apoptotic (e.g., Bad and Bax) and anti-apoptotic proteins (e.g., Bcl-2 and Bcl-xL), which control the process of apoptosis through release of caspases [32], [33]. Therefore, altered expression of the Bcl-2 family proteins observed upon honokiol treatment of pancreatic cancer cells in a manner that favors the increase in the ratios of Bax/Bcl-2 and Bax/Bcl-xL could underlie the observed apoptotic effect of honokiol. Deregulation of apoptosis-related proteins has also been reported in chondrosarcoma cells after honokiol treatment further supporting its role in apoptosis induction [34].

We also observed inhibition of NF-κB activity upon honokiol treatment, which was associated with inhibition of IκB-α phosphorylation and concomitant increase in its expression. Transcription factor NF-κB is constitutively activated in multiple malignancies and has been reported to be pathologically implicated in pancreatic cancer [18]–[20]. Recent studies have shown that the growth-suppressive effect of honokiol in prostate and colon cancers is mediated through the inhibition of NF-κB [35]. The NF-κB transcription factor is composed of heterodimers consisting of Rel (p65, c-Rel and RelB), p52, and p50 proteins and is localized in the cytoplasm in its inactive form in complex with IκB (inhibitor of NFκB consisting of α and β subunits that masks its nuclear localization signal [36]. Activation of NF-κB is caused, when IκB-α gets phosphorylated by the IKK (inhibitor kinase) complex leading to its ubiquitination and degradation. This results in the release of NFκB from the cytoplasm and transport into the nucleus followed by activation of NF-κB-responsive promoter. NF-κB is known to induce the expression of cyclin D1, Bcl-2 and Bcl-xL (altered upon honokiol treatment in current study) along with an array of proteins involved in cell proliferation and survival [37], [38]. Therefore, it can be suggested that growth suppression of pancreatic cancer cells by honokiol is mediated through the inhibition of NF-κB.

Clinical outcome in pancreatic cancer has remained poor due to advanced and metastatic state of the disease at the time of diagnosis and ineffectiveness of allowable drug-therapy due to chemoresistance [3], [5], [10]. Currently, gemcitabine is the national standard chemotherapeutic drug for pancreatic cancer treatment. Gemcitabine interferes with DNA synthesis leading to cell cycle arrest and apoptosis in ultimate course [39], [40]. One of the mechanisms that limits gemcitabine efficacy is induced activation of NF-κB in response to its treatment [41], which may cause apoptotic delay or suppression. In this study, we have shown chemosensitizing effect of honokiol on pancreatic cancer cells to gemcitabine toxicity. Additionally, we showed that gemcitabine treatment induced nuclear accumulation of NF-κB, which could be effectively inhibited by co-treatment with honokiol. These parallel findings indicate that the inhibition of NF-κB activity may also mediate the chemosensitization of pancreatic cancer cells by honokiol. Indeed, it has been reported earlier that anti-cancer effects of chemotherapeutic agents can be potentiated by inhibition of NF-κB activity [22], [41].

In conclusion, we have shown, for the first time, the growth inhibitory and chemosensitizing potential of honokiol in pancreatic cancer. Honokiol causes G_1_ phase cell cycle arrest and induction of apoptosis by altering the expression of cell cycle and survival associated proteins. Inhibition of NF-κB may be one of the significant mechanisms in honokiol-induced growth suppressive and chemosensitizing effects in pancreatic cancer cells. We therefore believe that honokiol could be a novel promising natural agent for the treatment of pancreatic cancer and may also serve as a chemosensitizer to improve the therapeutic efficacy of gemcitabine, which is already in clinical use as a therapeutic drug.

## Materials and Methods

### Reagents

Dulbecco's modified Eagle's medium (DMEM) and Roswell park memorial institute (RPMI-1640) medium were obtained from Thermo Scientific (Logan, UT). Fetal-bovine serum (FBS) was from Atlanta Biologicals (Lawrenceville, GA). Penicillin, streptomycin and trypsin-EDTA were purchased from Invitrogen (Carlsbad, CA). Honokiol was procured from LKT Laboratories (St. Paul, MN). Gemcitabine was provided by USAMCI pharmacy. FuGENE transfection reagent, phosphatase/protease inhibitors cocktail and cell proliferation reagent WST-1 were procured from Roche Diagnostics (Mannheim, Germany). Propidium iodide/RNAse staining buffer and PE Annexin V apoptosis detection kit were purchased from BD Bioscience (San Diego, CA). Nuclear extract kit was procured from Active Motif, LLC (Carlsbad, CA). Antibodies against Bcl-2, Bax and p-IκB-α (Ser32/36) (rabbit polyclonal), Bcl-xl and NF-κB/p65 (rabbit monoclonal), and IκB-α (mouse monoclonal) were obtained from Cell Signaling Technology (Beverly, MA). Antibodies against p21, Cdk4 (mouse monoclonal), p27, cyclin D1, cyclin E, Cdk2 (rabbit polyclonal), and horseradish peroxidase-conjugated secondary antibodies were procured from Santa Cruz Biotechnology (Santa Cruz, CA). β-Actin (mouse monoclonal) antibody was purchased from Sigma-Aldrich (St. Louis, MO). ECL plus western blotting detection kit was procured from Thermo Scientific. pGL4.32[luc2P/NF-κB -RE/Hygro] plasmid, pRL-TK plasmid and Dual Luciferase Assay System kit were from Promega (Madison, WI).

### Cell culture and treatments

The human pancreatic cell lines MiaPaCa and Panc1 (ATCC, Manassas, VA) were maintained in culture as adherent monolayer in RPMI-1640 and DMEM respectively, supplemented with 10% (v/v) FBS, penicillin (100 units/mL) and streptomycin (100 µg/mL). Cells were maintained in 5% CO_2_ humidified incubator at 37°C. Growth medium was changed every 3 day and cells were split (1∶3) when they reached 80% confluence. For treatments, stock solution of honokiol (10 mmol/L) was prepared in DMSO, stored at −20°C, and diluted with fresh complete medium immediately before use. Cells were treated with various concentrations of honokiol alone, gemcitabine alone or in combination (as specified in the figure legends). An equal volume of DMSO (final concentration, <0.1%) was added to the control.

### Cell growth assay

Cells were seeded in 96 well plates (1×10^4^ cells/well) a day prior to treatments. Cell viability in the treated cells was examined after 24–72 h by using WST-1 (4-[3-(4-iodophenyl)-2-(4-nitrophenyl)-2H-5-tetrazolio]-1, 3-benzene di-sulfonate) assay kit as per manufacturer's instructions with appropriate controls. This assay is based on the cleavage of WST-1 in metabolically active cells to form water-soluble formazan. The absorbance of the formazan was measured at a wavelength of 450 nm, with background subtraction at 630, using a Bio-Rad Benchmark microplate reader (Bio-Rad Laboratories, Hercules, CA). Growth was calculated as percent viability  =  [(A/B)×100], where A and B are the absorbance of treated and control cells, respectively.

### Cell-cycle analysis

The effect of honokiol treatment on cell cycle progression was determined by flow cytometry following staining with propidium iodide (PI). In brief, cells (1×10^6^ cells/well) were seeded in 6 well plate and synchronized by culturing them in serum free media. After 48 h, medium was replaced with complete medium containing desired concentrations of honokiol or DMSO. Floating and attached cells were collected after 24 h of treatment and fixed in 70% ethanol overnight at 4°C. The cells were then stained with propidium iodide, using PI/RNase staining buffer for 1 h at 37°C. Stained cells were analyzed by flow-cytometry on a BD-FACS Canto™ II (Becton-Dickinson, San Jose, CA) to calculate the percentage of cell population in various phases of cell cycle using Mod Fit LT software (Verity Software House, Topsham, ME).

### Apoptosis analysis

MiaPaCa and Panc1 cells were seeded (1×10^6^ cells/well) in 6 well plate. After overnight incubation, cells were treated with either control vehicle (DMSO) or various concentrations of honokiol for 24 h. Following treatment, cells were harvested, and stained with 7-Amino-Actinomycin (7-AAD) and PE Annexin V, using PE Annexin V Apoptosis Detection Kit I, followed by flow cytometry. Percentage of cell population in apoptosis was calculated using Mod Fit LT software.

### Nuclear and cytoplasmic fractionation

The preparation of cytoplasmic and nuclear extracts was performed using the Nuclear Extract Kit. In brief, cells were washed following treatment with 1 mL ice-cold PBS/phosphatase inhibitors, lysed in 500 µL hypotonic buffer and then centrifuged at 14,000 g for 30 s at 4°C. After collecting supernatant (cytoplasmic fraction), pellets were resuspended in 50 µL complete lysis buffer, and centrifuged at 14,000 g for 10 min at 4°C, and supernatant (nuclear fraction) were stored at −80°C.

### Western blot analysis

Cells were processed for protein extraction and western blotting as described earlier [10]. Immunodetection was carried out using specific antibodies: Bcl-2, Bcl-xL, Bax, NF-κB/p65, p-IκB-α (Ser32/36), IκB-α (1∶1000), Cdk2, Cdk4, Cyclin D1, Cyclin E, p21, p27 (1∶200) and β-actin (1∶20000). All respective secondary antibodies were used at 1∶2500 dilutions. Blots were processed with ECL plus Western Blotting detection kit and the signal detected using an LAS-3000 image analyzer (Fuji Photo Film Co., Tokyo, Japan). Densitometry was performed using an AlphaImager (Alpha Innotech Corp., San Leandro, CA).

### NF-κB transcriptional activity assay

To measure the NF-κB transcriptional activity, pancreatic cancer cells were seeded (0.5×10^6^ cells/well) in 12-well plate. After 60% confluence level, the cells were transiently transfected with 1 µg of NF-κB -luciferase promoter-reporter construct (pGL4.32 [luc2P/NF-κB -RE/Hygro]) and 0.5 µg of control reporter plasmid (pRL-TK), containing Renilla reniformis luciferase gene downstream of the TK promoter. Transfections were carried out using FuGENE as a transfection reagent according to the manufacturers' recommendations. Twenty-four hours after transfection, the cells were treated with honokiol for next 24 h, washed with ice cold PBS, and harvested in reporter lysis buffer. Luciferase activity was measured using the Dual Luciferase Assay System. All experiments were carried out in triplicate and relative luciferase activity reported as the fold induction after normalization for transfection efficiency.

### Statistical analysis

All the experiments were performed at least three times, independently. A logistic regression model was fit to the data using R statistical software to calculate the IC_50_. The data obtained were expressed as ‘mean ± standard deviation’. Wherever appropriate, the data were also subjected to unpaired two tailed Student's t-test. A value of p<0.05 was considered as significant.

## References

[1] Jemal A, Siegel R, Ward E, Hao Y, Xu J (2009). Cancer statistics, 2009.. CA Cancer J Clin.

[2] Jemal A, Siegel R, Xu J, Ward E (2010). Cancer statistics, 2010.. CA Cancer J Clin.

[3] Wong HH, Lemoine NR (2009). Pancreatic cancer: molecular pathogenesis and new therapeutic targets.. Nat Rev Gastroenterol Hepatol.

[4] Singh AP, Moniaux N, Chauhan SC, Meza JL, Batra SK (2004). Inhibition of MUC4 expression suppresses pancreatic tumor cell growth and metastasis.. Cancer Res.

[5] Olive KP, Jacobetz MA, Davidson CJ, Gopinathan A, McIntyre D (2009). Inhibition of Hedgehog signaling enhances delivery of chemotherapy in a mouse model of pancreatic cancer.. Science.

[6] Li D, Xie K, Wolff R, Abbruzzese JL (2004). Pancreatic cancer.. Lancet.

[7] Dhayat S, Mardin WA, Mees ST, Haier J (2011). Epigenetic markers for chemosensitivity and chemoresistance in pancreatic cancer - A review.. Int J Cancer.

[8] Giovannetti E, Funel N, Peters GJ, Del CM, Erozenci LA (2010). MicroRNA-21 in pancreatic cancer: correlation with clinical outcome and pharmacologic aspects underlying its role in the modulation of gemcitabine activity.. Cancer Res.

[9] Yokoi K, Fidler IJ (2004). Hypoxia increases resistance of human pancreatic cancer cells to apoptosis induced by gemcitabine.. Clin Cancer Res.

[10] Singh S, Srivastava SK, Bhardwaj A, Owen LB, Singh AP (2010). CXCL12-CXCR4 signalling axis confers gemcitabine resistance to pancreatic cancer cells: a novel target for therapy.. Br J Cancer.

[11] Newman DJ, Cragg GM, Snader KM (2003). Natural products as sources of new drugs over the period 1981-2002.. J Nat Prod.

[12] Gupta SC, Kim JH, Prasad S, Aggarwal BB (2010). Regulation of survival, proliferation, invasion, angiogenesis, and metastasis of tumor cells through modulation of inflammatory pathways by nutraceuticals.. Cancer Metastasis Rev.

[13] Chen F, Wang T, Wu YF, Gu Y, Xu XL (2004). Honokiol: a potent chemotherapy candidate for human colorectal carcinoma.. World J Gastroenterol.

[14] Li Z, Liu Y, Zhao X, Pan X, Yin R (2008). Honokiol, a natural therapeutic candidate, induces apoptosis and inhibits angiogenesis of ovarian tumor cells.. Eur J Obstet Gynecol Reprod Biol.

[15] Chilampalli S, Zhang X, Fahmy H, Kaushik RS, Zeman D (2010). Chemopreventive effects of honokiol on UVB-induced skin cancer development.. Anticancer Res.

[16] Park EJ, Min HY, Chung HJ, Hong JY, Kang YJ (2009). Down-regulation of c-Src/EGFR-mediated signaling activation is involved in the honokiol-induced cell cycle arrest and apoptosis in MDA-MB-231 human breast cancer cells.. Cancer Lett.

[17] Yang SE, Hsieh MT, Tsai TH, Hsu SL (2002). Down-modulation of Bcl-XL, release of cytochrome c and sequential activation of caspases during honokiol-induced apoptosis in human squamous lung cancer CH27 cells.. Biochem Pharmacol.

[18] Basseres DS, Baldwin AS (2006). Nuclear factor-kappaB and inhibitor of kappaB kinase pathways in oncogenic initiation and progression.. Oncogene.

[19] Liptay S, Weber CK, Ludwig L, Wagner M, Adler G (2003). Mitogenic and antiapoptotic role of constitutive NF-kappaB/Rel activity in pancreatic cancer.. Int J Cancer.

[20] Wharry CE, Haines KM, Carroll RG, May MJ (2009). Constitutive non-canonical NFkappaB signaling in pancreatic cancer cells.. Cancer Biol Ther.

[21] Dahlman JM, Wang J, Bakkar N, Guttridge DC (2009). The RelA/p65 subunit of NF-kappaB specifically regulates cyclin D1 protein stability: implications for cell cycle withdrawal and skeletal myogenesis.. J Cell Biochem.

[22] Kamat AM, Sethi G, Aggarwal BB (2007). Curcumin potentiates the apoptotic effects of chemotherapeutic agents and cytokines through down-regulation of nuclear factor-kappaB and nuclear factor-kappaB-regulated gene products in IFN-alpha-sensitive and IFN-alpha-resistant human bladder cancer cells.. Mol Cancer Ther.

[23] Ghosh S, Karin M (2002). Missing pieces in the NF-kappaB puzzle.. Cell.

[24] Hanahan D, Weinberg RA (2011). Hallmarks of cancer: the next generation.. Cell.

[25] Call JA, Eckhardt SG, Camidge DR (2008). Targeted manipulation of apoptosis in cancer treatment.. Lancet Oncol.

[26] Kastan MB, Bartek J (2004). Cell-cycle checkpoints and cancer.. Nature.

[27] Vermeulen K, Van Bockstaele DR, Berneman ZN (2003). The cell cycle: a review of regulation, deregulation and therapeutic targets in cancer.. Cell Prolif.

[28] Satyanarayana A, Kaldis P (2009). Mammalian cell-cycle regulation: several Cdks, numerous cyclins and diverse compensatory mechanisms.. Oncogene 20;.

[29] Abukhdeir AM, Park BH (2008). P21 and p27: roles in carcinogenesis and drug resistance.. Expert Rev Mol Med.

[30] Fong WF, Tse AK, Poon KH, Wang C (2005). Magnolol and honokiol enhance HL-60 human leukemia cell differentiation induced by 1,25-dihydroxyvitamin D3 and retinoic acid.. Int J Biochem Cell Biol.

[31] Chen CY, Hsu YL, Chen YY, Hung JY, Huang MS (2007). Isokotomolide A, a new butanolide extracted from the leaves of Cinnamomum kotoense, arrests cell cycle progression and induces apoptosis through the induction of p53/p21 and the initiation of mitochondrial system in human non-small cell lung cancer A549 cells.. Eur J Pharmacol.

[32] Fong WF, Tse AK, Poon KH, Wang C (2005). Magnolol and honokiol enhance HL-60 human leukemia cell differentiation induced by 1,25-dihydroxyvitamin D3 and retinoic acid.. Int J Biochem Cell Biol.

[33] Karin M (2006). Nuclear factor-kappaB in cancer development and progression.. Nature.

[34] Chen YJ, Wu CL, Liu JF, Fong YC, Hsu SF (2010). Honokiol induces cell apoptosis in human chondrosarcoma cells through mitochondrial dysfunction and endoplasmic reticulum stress.. Cancer Lett.

[35] Lee SY, Yuk DY, Song HS, Yoon dY, Jung JK (2008). Growth inhibitory effects of obovatol through induction of apoptotic cell death in prostate and colon cancer by blocking of NF-kappaB.. Eur J Pharmacol.

[36] Gilmore TD (2006). Introduction to NF-kappaB: players, pathways, perspectives.. Oncogene.

[37] Perkins ND (2000). The Rel/NF-kappa B family: friend and foe.. Trends Biochem Sci.

[38] Srivastava SK, Singh SV (2004). Cell cycle arrest, apoptosis induction and inhibition of nuclear factor kappa B activation in anti-proliferative activity of benzyl isothiocyanate against human pancreatic cancer cells.. Carcinogenesis.

[39] Cappella P, Tomasoni D, Faretta M, Lupi M, Montalenti F (2001). Cell cycle effects of gemcitabine.. Int J Cancer.

[40] Huang P, Plunkett W (1995). Fludarabine- and gemcitabine-induced apoptosis: incorporation of analogs into DNA is a critical event.. Cancer Chemother Pharmacol.

[41] Uwagawa T, Chiao PJ, Gocho T, Hirohara S, Misawa T (2009). Combination chemotherapy of nafamostat mesilate with gemcitabine for pancreatic cancer targeting NF-kappaB activation.. Anticancer Res.

